# Highly Sensitive and Temperature‐Insensitive Stretchable Strain Sensor with Microcrack‐Engineered Architecture

**DOI:** 10.1002/advs.202521634

**Published:** 2026-01-18

**Authors:** Yu Kato, Kento Yamagishi, Kenjiro Fukuda, Takao Someya, Tomoyuki Yokota

**Affiliations:** ^1^ Department of Electrical Engineering and Information Systems The University of Tokyo Bunkyo‐ku Tokyo Japan; ^2^ RIKEN Center for Emergent Matter Science Wako Saitama Japan; ^3^ RIKEN Thin‐Film Device Laboratory Wako Saitama Japan; ^4^ Graduate School of Engineering The University of Osaka Suita Osaka Japan; ^5^ Institute of Engineering Innovation Graduate School of Engineering The University of Tokyo Bunkyo‐ku Tokyo Japan

**Keywords:** conductive polymers, flexible electronics, microcracks, strain sensors, temperature coefficient of resistance

## Abstract

Highly sensitive, stretchable strain sensors with minimal temperature dependence are essential for precise strain monitoring in human and robotic applications across diverse environments. Although strain sensors with near‐zero temperature coefficient of resistance (TCR) have been investigated, achieving high sensitivity while maintaining temperature insensitivity remains a significant challenge. This study proposes a stretchable strain sensor that demonstrates a high gauge factor of 2.3 × 10^4^ and excellent linearity (*R* ^2^ = 0.98), while maintaining temperature insensitivity under applied strain. This performance is achieved through a microcrack architecture formed by stacking a brittle, low‐resistance material with a stretchable, high‐resistance material with near‐zero TCR. The formation of microcracks redirects the current pathway from a low to a high‐resistance material. The absolute TCR value remains below 0.1% K^−1^ across a strain range of 5%–50%. Furthermore, the peak resistance under 50% cyclic strain fluctuates by only 7% between 25°C and 65°C.

## Graphical abstract

This study proposes a stretchable strain sensor that demonstrates a high gauge factor of 2.3 × 10^4^ and excellent linearity (*R* ^2^ = 0.98), while maintaining temperature insensitivity under applied strain. This performance is achieved through a microcrack architecture formed by stacking a brittle, low‐resistance material with a stretchable, high‐resistance material with a near‐zero temperature coefficient of resistance (TCR).

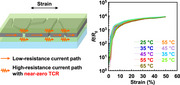

## 1 Introduction

Flexible strain sensors are widely utilized for monitoring human and robotic motion. By attaching these sensors to the body, a broad range of physiological and kinematic information can be captured, including limb and finger movements [1, 2, 3], facial expressions [4, 5], eye movements [6], respiration [7, 8, 9], swallowing [3, 10], speech [11, 12], and pulse signals [13, 14]. These capabilities have enabled significant advancements in fields such as medicine, healthcare, sports, and emerging applications within the metaverse. For wearable applications, strain sensors need to accommodate stretchability of up to 55% [15]. In robotics, both rigid and soft robots employ strain sensors to monitor joint articulation [16, 17] and the displacement of soft actuators [18, 19, 20], necessitating sensors that combine high elasticity with mechanical durability. Furthermore, high strain sensitivity is essential for the precise detection of subtle body and robotic movements.

Strain sensors are generally classified into resistive, capacitive, and piezoelectric types. Among these, resistive strain sensors are notable for their high sensitivity, achieved through the use of materials that demonstrate pronounced resistance variations under strain [21]. Various sensor materials have been explored for resistive strain sensors, such as carbon nanotubes [22], graphene [23], carbon black [24], Ag nanoparticles (NPs) [25], Ag nanowires [3], indium tin oxide [26], poly(3,4‐ethylenedioxythiophene):poly(styrenesulfonate) (PEDOT:PSS) [27], MXene [28], liquid metal [29], and hydrogels [30], often integrated with elastomers to impart stretchability. To further enhance both sensitivity and stretchability, the incorporation of controlled cracks within the sensor materials has been investigated. For example, crack‐based strain sensors have been developed using single‐layer [25, 28, 31, 32], multilayer [33, 34, 35], and bridge structures incorporating conductive fillers [14, 36].

A significant challenge associated with resistive strain sensors is their susceptibility to temperature fluctuations, as the temperature coefficient of resistance (TCR) for most conductors deviates from zero. This temperature dependence can compromise the accuracy of strain measurements across various temperatures [37]. To address this issue, research has focused on the development of strain sensors with near‐zero TCR. One effective strategy involves combining materials with positive and negative TCRs to offset the TCR [37, 38, 39, 40, 41, 42, 43, 44]. Furthermore, optimizing grains or island structures of sensor materials and precisely controlling charge transport properties can effectively minimize temperature dependence [45, 46, 47]. Specifically, secondary doping of PEDOT:PSS with dimethyl sulfoxide (DMSO) can shift the charge transport mechanism from variable‐range hopping to band‐like transport, thereby altering the TCR from negative to positive [45]. By fine‐tuning the DMSO concentration, a near‐zero TCR can be achieved. Other approaches to suppress the temperature dependence of flexible strain sensors include controlling thermal expansion [13], ligand exchange in Ag NPs [48], and the use of alloys [49]. Furthermore, strain sensors based on liquid metals [16, 29], hydrogels [50], and eutectogels [2] have demonstrated low temperature dependence.

However, a stretchable strain sensor that combines temperature insensitivity under strain with high strain sensitivity has yet to be achieved (Table S1) [2, 10, 16, 29, 38, 39, 40, 41, 50, 51, 52, 53, 54]. Even when the TCR is optimized in the initial state, the charge transport properties of the device can change as strain increases, resulting in a temperature dependence of resistance under strain. Furthermore, composite materials often demonstrate nonlinearity owing to strain‐induced changes in resistivity. For example, although some composite‐based sensors demonstrate high maximum gauge factors, their gauge factors in the linear range from 0% strain are typically limited to the tens, and their performance under cyclic loading exhibits slight susceptibility to temperature variations [38, 39, 40, 41]. Conversely, strain sensors with excellent stretchability and minimal strain‐dependent characteristics generally avoid these issues, but tend to display low gauge factors. For example, previous studies employing liquid metals, hydrogels, and kirigami structures have achieved wide working strain ranges, yet their gauge factors remain below 5 [16, 29, 50, 54]. Consequently, achieving both temperature insensitivity under strain and excellent strain sensitivity with a large gauge factor and linearity has remained a significant challenge.

This study introduces a stretchable strain sensor that achieves a high gauge factor of 2.3 × 10^4^ with excellent linearity, while maintaining temperature insensitivity under strain. This performance is achieved by engineering a microcrack structure by stacking a brittle, low‐resistance material with a stretchable, high‐resistance material with a near‐zero TCR. Upon straining, the formation of microcracks induces a substantial change in resistance by redirecting the current path from the low to the high‐resistance material. The device demonstrates a coefficient of determination of 0.98 for linearity within the 0%–50% strain range. A composite of polyurethane (PU) and PEDOT:PSS was employed as the stretchable high‐resistance material with near‐zero TCR. Incorporation of 10 wt% DMSO to PU‐PEDOT:PSS effectively tuned the TCR of PU‐PEDOT:PSS from negative to near‐zero values. The absolute TCR of the multilayer strain sensor remained below 0.1% K^−1^ across strains from 5% to 50%, validating temperature insensitivity during elongation, particularly when the electrical properties of the PU‐PEDOT:PSS layer dominated. Under cyclic strains ranging from 0% to 50% and temperatures between 25°C and 65°C, the peak resistance variation was limited to 7%.

## 2 Results

### 2.1 Multilayer Strain Sensors using a Material with Near‐Zero TCR

**FIGURE 1:**
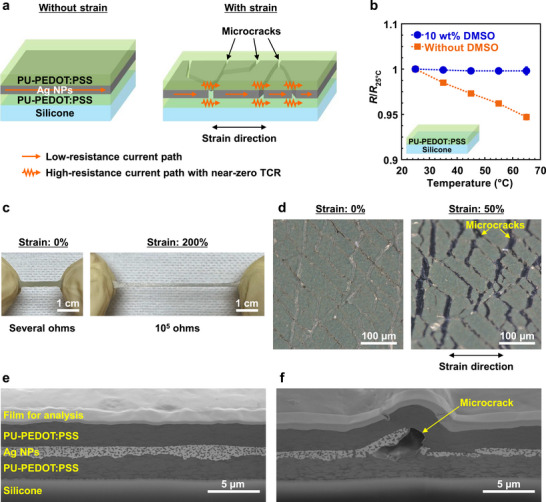
Materials and structures of multilayer strain sensors. a) Schematic cross‐section of the sensor. b) Temperature‐dependent resistance of PU‐PEDOT:PSS single‐layer structure under 50% strain. The error bars in the plots indicate standard deviations based on four devices. c) Images of the sensor in the unstrained state and under approximately 200% strain. d) Optical microscopic images of the sensor in the unstrained state and under 50% strain observed from the surface. Strain was applied horizontally in the image. e, f) Cross‐sectional SEM images of the multilayer strain sensor in the unstrained state. Areas without microcracks (e) and with a microcrack (f). c–f) 50% strain was applied to the device before the analysis to form microcracks. DMSO concentration was 10 wt% for these devices.

The schematic cross‐section of the strain sensor is shown in Figure 1a. A multilayer structure was used in which conductive layers of PU‐PEDOT:PSS, Ag NPs, and PU‐PEDOT:PSS were sequentially stacked on a silicone elastomer substrate through spin‐coating (Figure S1). DMSO was added to the PU‐PEDOT:PSS ink to control its properties. In this configuration, Ag NPs functioned as a brittle and low‐resistance material, whereas PU‐PEDOT:PSS served as a stretchable and high‐resistance material with near‐zero TCR. In the absence of applied strain, the device resistance was low owing to the conductive path of low‐resistance Ag. Upon application of strain, microcracks formed within the brittle Ag NPs, locally disrupting the Ag current pathways. Consequently, the continuous PU‐PEDOT:PSS layer became the primary current path, resulting in an increase in device resistance. This mechanism is expected to yield a high gauge factor for the multilayer strain sensor. Furthermore, because the device resistance under strain was predominantly governed by the PU‐PEDOT:PSS layer with near‐zero TCR, the temperature dependence of the device under strain could be suppressed.

The addition of DMSO to PEDOT:PSS enhances its electrical conductivity and shifts the TCR positively [45]. As shown in Figure S2, the conductivity of PU‐PEDOT:PSS increased with DMSO incorporation. The temperature‐dependent resistance of PU‐PEDOT:PSS, with *R*_25°C_ representing the resistance at 25°C, is shown in Figure 1b. Without DMSO, PU‐PEDOT:PSS displayed a negative TCR characterized by a decrease in resistance at elevated temperatures. In contrast, the addition of 10 wt% DMSO resulted in a near‐zero TCR, with minimal resistance variation across the temperature range.

Images of the multilayer strain sensor are shown in Figure 1c. The sensor demonstrates excellent stretchability, sustaining strains up to 200% without mechanical failure. The sensor structure was further analyzed after subjecting the device to 50% strain. The optical microscopic images of the sensor observed from the surface are shown in Figure 1d, revealing numerous microcracks. In the unstrained state, these microcracks remained closed, whereas the application of strain caused them to open. As shown in Figure S3, the crack length increases proportionally with applied strain. A cross‐sectional scanning electron microscopy (SEM) image of an area without microcracks is shown in Figure 1e, where PU‐PEDOT:PSS and Ag NPs formed continuous films in each layer. In Figure S4a,b, a mesh‐like contrast was observed within the PU‐PEDOT:PSS, which corresponds to the sulfur distribution identified by energy dispersive X‐ray spectroscopy (EDX) elemental mapping shown in Figure S4c. This indicates that the sulfur‐containing PEDOT:PSS formed a network structure within the PU matrix, a configuration that is likely advantageous for maintaining electrical conductivity under strain. An SEM image of an area with a microcrack is shown in Figure 1f. The microcrack is confined to the Ag NP layer and does not extend into the PU‐PEDOT:PSS. This can be attributed to the properties of the PU (SUPERFLEX E‐2000, DKS Co. Ltd.), which is a soft material with a low Young's modulus of 11 MPa, rendering it resistant to fracture. During stretching, significant strain was localized in the PU‐PEDOT:PSS near the microcrack in Ag NPs, resulting in local delamination at the interface between the PU‐PEDOT:PSS and Ag NPs. Upon release of the strain, the PU‐PEDOT:PSS formed a convex shape owing to localized plastic deformation. Importantly, the PU‐PEDOT:PSS layer remains continuous, thereby providing a conductive path near the microcrack in the multilayer structure.

### 2.2 Differences in Characteristics Between Single‐Layer and Multilayer Structures

**FIGURE 2:**
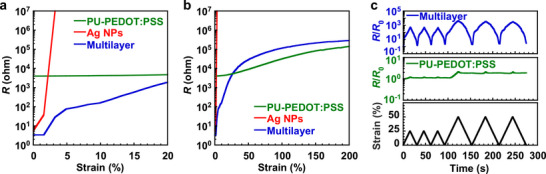
Resistance–strain characteristics of single‐layer and multilayer structures at room temperature. a, b) Resistance of devices as a function of applied strain. Strain ranges from 0% to 20% (a) and 0% to 200% (b). c) Relative resistance variations in the PU‐PEDOT:PSS single‐layer and multilayer structures to cyclic strain. a–c) DMSO concentration was 10 wt%.

The characteristics of the multilayer structures were systematically compared with those of the single‐layer structures. The resistance of various device architectures (Figure S5) was evaluated under dynamic strain. The resistance change of devices with respect to strain is shown in Figure 2a,b. In the absence of strain, the multilayer structure demonstrated low resistance, comparable with that of the Ag NP single‐layer structure (Figure 2a; Figure S6). In contrast, the PU‐PEDOT:PSS single‐layer structure demonstrated a resistance three orders of magnitude higher than that of the other structures without strain. Upon application of strain, the Ag NP single‐layer structure became insulated at strains below 5%. Conversely, both the PU‐PEDOT:PSS single‐layer and multilayer structures maintained excellent stretchability, with resistance remaining below 1 Mohms even at strains up to 200%. Notably, at strains exceeding 30%, the resistance of the multilayer structure approached that of the PU‐PEDOT:PSS single‐layer structure. This behavior suggests that the low‐resistance current pathways provided by the Ag layer were largely disrupted owing to the formation of microcracks under strain. The electrical conduction in the multilayer structure probably shifted from Ag‐based to PU‐PEDOT:PSS‐based pathways with strain. Furthermore, the multilayer structure demonstrated higher strain sensitivity compared with the PU‐PEDOT:PSS single‐layer structure, attributable to the influence of microcrack formation.

Relative resistance variations in the PU‐PEDOT:PSS single‐layer and multilayer structures to cyclic strain are shown in Figure 2c. The PU‐PEDOT:PSS single‐layer structure indicated a minor, irreversible change in resistance to strain, a well‐documented limitation of elastomer/PEDOT:PSS composites [27, 55]. Owing to this irreversible behavior, the PU‐PEDOT:PSS single‐layer structure is unsuitable for reliable strain‐sensing applications. In contrast, the multilayer structure demonstrated a significant and reversible change in resistance under applied strain. Specifically, after repeated cycles at 50% strain, the multilayer sensor demonstrated a resistance change exceeding three orders of magnitude, whereas the single‐layer structure showed a maximum change of only 20%. These findings highlight the superior stretchability, strain sensitivity, and reversibility achieved with the multilayer structure.

### 2.3 Characteristics of Multilayer Strain Sensors

**FIGURE 3:**
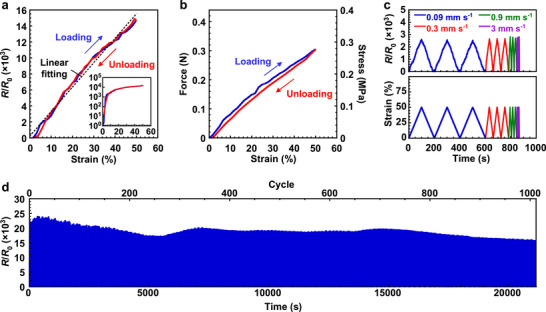
Characteristics of multilayer strain sensors at room temperature. a) Relative resistance change as a function of applied strain. The inset shows a figure with a logarithmic scale on the vertical axis. b) Force and stress–strain characteristics. Stress was calculated based on the total thickness of the device. c) Relative resistance variation at different strain rates under 50% cyclic strain. d) Durability of the sensor under cyclic strains ranging from 0% to 50% for approximately 1000 cycles. a–d) 50% strain was applied at 70°C before the evaluation. DMSO concentration was 10 wt%.

Characteristics of multilayer strain sensors under repeated strains of up to 50% were evaluated at room temperature, with stabilization achieved by applying 50% strain at 70°C. The gauge factor of the strain sensors is expressed as follows:

(1) $$\mathrm{Gauge}\;\mathrm{factor}=\frac{\Delta R}{R_{0}}\frac{1}{\varepsilon }$$

where Δ*R* represents the change in resistance owing to strain, *R*_0_ represents the initial resistance, and *ε* represents the strain. In this study, the gauge factor was calculated at a strain of 50%. As shown in Figure 3a, the multilayer strain sensor demonstrated a highly linear and consistent resistance response during both loading and unloading cycles. The variation in sensor characteristics is shown in Figure S7. Across seven devices, the average gauge factor was 2.3 × 10^4^ with a standard deviation of 1.1 × 10^4^, and the average coefficient of determination for linearity was 0.98 with a standard deviation of 0.01. The force and stress–strain characteristics are shown in Figure 3b. Owing to the excellent elasticity of the silicone elastomer employed as a substrate, the sensor displayed reversible mechanical properties. The relative resistance variation at different strain rates is shown in Figure 3c, indicating minimal dependence of resistance change on strain rate. The response and recovery times of multilayer strain sensors are shown in Figure S8. The response (*t*_res_) and recovery (*t*_rec_) times were defined as the durations required for resistance fluctuations to stabilize to less than 3% s^−1^ after strain fixation. The measured response and recovery times were 4.6 and 2.8 s, respectively, with resistance drift subsiding quickly.

The cyclic durability of the sensor under repeated loading is shown in Figure 3d and Figure S9. The sensor was subjected to cyclic strains ranging from 0% to 50% for approximately 1000 cycles. Throughout this process, the sensor consistently displayed distinct resistance responses to applied strain, even after 1000 cycles. The peak resistance decreased slightly by 8% after 100 cycles and by 31% after 1000 cycles. Previous studies have reported that in PU‐PEDOT:PSS composites, the PEDOT:PSS network within the PU matrix can be disrupted and reformed during repeated strain [55], which likely contributes to the observed resistance fluctuation in this study. After 1000 cycles, the sensor was subjected to 50% strain at 70°C, followed by performance evaluation at room temperature (Figure S9d). Under these conditions, the decrease in peak resistance from the initial cycle was limited to 11%. These results demonstrated that the sensor was durable for over 1000 cycles.

### 2.4 TCR under Static Strain

**FIGURE 4:**
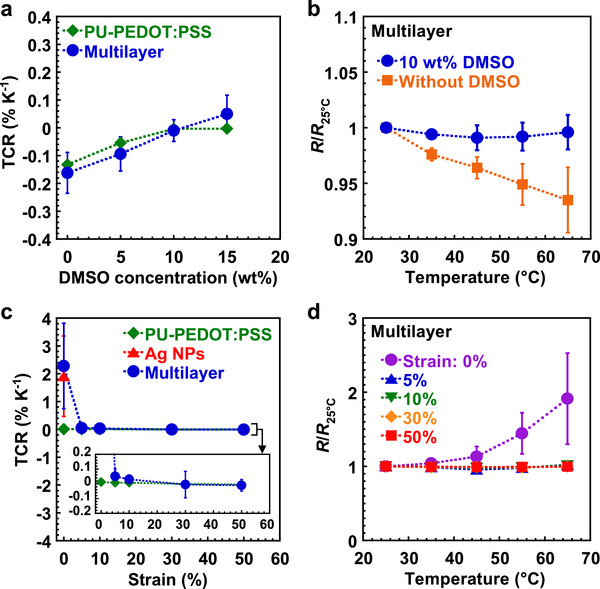
TCR and temperature dependence of devices under static strain. a) Relationship between TCR and DMSO concentration in PU‐PEDOT:PSS single‐layer and multilayer structures under 50% strain. b) Relative resistance variation in multilayer structures with temperature under 50% strain. c) Relationship between TCR and strain in different structures. The inset shows a magnified view of the vertical axis. d) Relative resistance variation in multilayer structures with temperature at each strain. c, d) DMSO concentration was 10 wt%. a–d) The error bars in the plots indicate standard deviations based on three or four devices.

To evaluate the TCR at each strain, we measured the resistance of the devices as a function of temperature while maintaining constant strain. The TCR is expressed as follows:

(2) $$\mathrm{TCR}=\frac{\Delta R}{R_{0}}\frac{1}{\left(T_{1}-T_{0}\right)}$$

where Δ*R* represents the difference in resistance at temperatures *T*_1_ and *T*_0_, and *R*_0_ represents the initial resistance at temperature *T*_0_. We calculated the TCR from the resistance at 25°C and 65°C. The relationship between TCR and DMSO concentration in PU‐PEDOT:PSS single‐layer and multilayer structures under 50% strain is shown in Figure 4a. In the multilayer structure, increasing the DMSO concentration resulted in a positive shift in TCR, consistent with the trend observed in the PU‐PEDOT:PSS single‐layer structure. This indicates that DMSO incorporation modifies the TCR of the PU‐PEDOT:PSS layer, which in turn influences the overall TCR of the multilayer structure under strain because the device resistance under strain was predominantly governed by the PU‐PEDOT:PSS layer. Specifically, the TCR of the multilayer structure shifted from −0.16% K^−1^ without DMSO, to −0.01% K^−1^ with the addition of 10 wt% DMSO, effectively transitioning from a negative to near‐zero TCR. The relative resistance change with temperature for multilayer structures with and without DMSO is shown in Figure 4b. *R*_25°C_ defines the resistance at 25°C. In the absence of DMSO, the resistance decreased by 6.5% as the temperature increased from 25°C to 65°C. Conversely, the device containing 10 wt% DMSO demonstrated less than 1% change in resistance over the same temperature range. These results demonstrate that the addition of an optimal concentration of DMSO to PU‐PEDOT:PSS effectively suppresses the temperature dependence of resistance under strain.

The relationship between TCR and strain in different device structures is shown in Figure 4c. Both the single‐layer and multilayer configurations incorporated 10 wt% DMSO into the PU‐PEDOT:PSS matrix. In the unstrained state, the multilayer structure displayed a TCR of 2.3% K^−1^, comparable with that of the Ag NP single‐layer structure. However, under applied strains ranging from 5% to 50%, the absolute TCR value of the multilayer structure decreased to less than 0.1% K^−1^, aligning closely with that of the PU‐PEDOT:PSS single‐layer structure. The TCR of the multilayer structure was influenced by different layers depending on the applied strain. The relative resistance variation in multilayer structures with temperature at each strain is shown in Figure 4d. In the absence of strain, the resistance increased by a factor of 1.9 as the temperature rose from 25°C to 65°C. In contrast, when subjected to strains between 5% and 50%, the resistance variation between 25°C and 65°C remained within 2%. This demonstrates that the temperature dependence of the multilayer structure under strain was suppressed owing to PU‐PEDOT:PSS with near‐zero TCR.

### 2.5 Strain Sensor Characteristics at Different Temperatures

**FIGURE 5:**
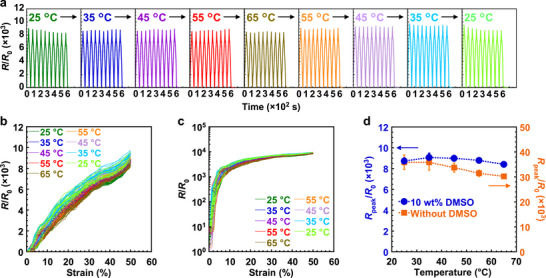
Characteristics of multilayer strain sensors at different temperatures under cyclic strain. a) Relative resistance change for 10 cycles of 50% cyclic strain at each temperature. b, c) Relative resistance change as a function of strain. Figures with linear scale (b) and logarithmic scale (c) on the vertical axis. a–c) DMSO concentration was 10 wt%. d) Relationship between peak resistance and temperature for devices with 10 wt% DMSO and without DMSO. The error bars in the plots indicate standard deviations based on characteristics of 20 or 10 cycles. a–d) *R*_0_ denotes the resistance of the device without strain at 25°C. 50% strain was applied at 70°C before the evaluation.

The strain sensor performance was evaluated under cyclic strain at different temperatures. Prior to testing, a 50% strain was applied at 70°C to stabilize the device. The relative resistance change of the multilayer strain sensor is shown in Figure 5a, with resistance values normalized to the unstrained resistance at 25°C. The sensor demonstrated minimal variation in resistance response to 50% cyclic strain across different temperatures. The relative resistance variations of the sensor as a function of strain are shown in Figure 5b,c. As illustrated, the data from Figure 5a are reorganized with strain as the horizontal axis. The strain sensor demonstrated minimal variation in its characteristics as the temperature changed from 25°C to 65°C. For comparison, the temperature dependence of a multilayer strain sensor without DMSO is shown in Figure S10. In the absence of DMSO, the resistance response to the strain diminished at higher temperatures. The relationship between peak resistance (*R*_peak_) and temperature is shown in Figure 5d. For the device without DMSO, *R*_peak_ decreased with increasing temperature, consistent with the negative TCR of PU‐PEDOT:PSS without DMSO. The difference in peak resistance between temperatures was 16%. In contrast, the device incorporating 10 wt% DMSO demonstrated only a 7% difference in peak resistance, demonstrating significantly reduced temperature sensitivity.

A summary of the performance of temperature‐insensitive stretchable strain sensors is provided in Table S1 [2, 10, 16, 29, 38, 39, 40, 41, 50, 51, 52, 53, 54]. In this study, a high gauge factor with excellent linearity was achieved by leveraging the opening and closing of microcracks within the multilayer structure, while temperature insensitivity under strain was maintained through the use of DMSO‐doped PU‐PEDOT:PSS with a near‐zero TCR.

## 3 Discussion

The multilayer strain sensor demonstrated a linear sensitivity to applied strain. In contrast, many composite materials typically demonstrate nonlinear sensitivity owing to variations in resistivity under strain. The underlying mechanism governing resistance changes in multilayer structures with strain differs fundamentally from that of composite materials. In the multilayer configuration, resistance modulation resulted from alterations in the electrical conduction path between Ag and PU‐PEDOT:PSS associated with the opening and closing of microcracks. Under strain, electrical current is presumed to traverse the high‐resistance PU‐PEDOT:PSS regions adjacent to these microcracks. As shown in Figure S3b, the crack length increased almost proportionally to the strain, suggesting that the length of the current path in PU‐PEDOT:PSS near the microcracks increased proportionally to the strain. Because the device resistance under strain is primarily governed by the resistance of PU‐PEDOT:PSS, the overall device resistance is expected to increase linearly as the crack length increases linearly with strain. Therefore, the multilayer strain sensor, which operates based on the microcrack dynamics, is particularly effective in achieving linear sensitivity.

The properties of PU‐PEDOT:PSS were further enhanced by secondary doping with DMSO, which shifted its TCR from negative to near‐zero values (Figure 4a). In aqueous dispersion, PEDOT:PSS forms a micelle structure in which conductive PEDOT is encapsulated by insulating PSS [56]. Secondary doping with a polar solvent induces crystallization of PEDOT and promotes phase separation between PEDOT and PSS, thereby enhancing the electrical conductivity of PEDOT:PSS [45, 56, 57]. Moreover, DMSO doping shifts the TCR in a positive direction, attributed to a transition in the carrier transport mechanism from variable‐range hopping transport to band‐like transport [45]. Our results demonstrated that the incorporation of DMSO to PU‐PEDOT:PSS—a composite of PU and PEDOT:PSS—not only enhanced conductivity (Figure S2) but also resulted in a positive shift in TCR (Figure 4a). This observation suggests that the TCR modulation in PU‐PEDOT:PSS occurred through a mechanism analogous to that previously reported for PEDOT:PSS [45]. In this study, we achieved stretchable materials capable of maintaining a near‐zero TCR even during elongation by blending with PU, thereby enabling the development of temperature‐insensitive stretchable strain sensors.

As shown in Figure 4c, the TCR of the multilayer structure in the unstrained state was measured at 2.3% K^−1^, notably higher than that of bulk silver (0.38% K^−1^) [58]. Generally, the resistance change in such devices with temperature is influenced not only by the TCR of the sensor materials but also by thermal expansion effects [59, 60, 61]. This relationship can be expressed as follows:

(3) $$\frac{\Delta R}{R_{0}}=\alpha_{\mathrm{mat}}\Delta T+K\beta \Delta T$$

where *α*_mat_ represents the TCR of sensor materials associated with charge transport behavior, Δ*T* represents the change in temperature, *K* represents the gauge factor, and *β* represents the coefficient of thermal expansion (CTE). By dividing both sides by ΔT, the equation can be reformulated as:

(4) $$\frac{\Delta R}{R_{0}}\frac{1}{\Delta T}=\alpha_{\mathrm{mat}}+K\beta$$

Therefore, the device TCR is determined by both the intrinsic temperature dependence of sensor materials and the piezoresistive effect resulting from the product of the CTE and gauge factor. For reference, the CTE of silicone elastomer (SYLGARD 184, Dow Corning Inc.) is reported to be 266.5 ppm°C^−1^ [62]. The gauge factor of the multilayer strain sensors near 0% strain, calculated over the 0%–1.6% strain range (Figure S7), had a median value of 140. Therefore, *Kβ* was calculated to be 3.7% K^−1^, indicating that thermal expansion influenced the TCR of the device. This temperature dependency poses a challenge for applications requiring the measurement of small strains below 5%, whereas for human applications, strain sensors are required to measure large strains of several tens of percent [15]. This temperature dependency can be mitigated by selecting elastomers with low CTE.

The multilayer structural approach presented here effectively enables simultaneous control over both strain sensitivity and temperature insensitivity in strain sensors. Sensors engineered for near‐zero TCR can accurately detect human motion by minimizing the effects of ambient and body temperature fluctuations [37] and are suitable for use in contact with hot or cold objects [63]. This strategy is also effective for machines with significant fluctuations in the environmental temperature. The movements of a robot's joint are sensed using a temperature‐insensitive strain sensor [16]. We anticipate that the sensors developed in this study will find broad application in wearable devices and robotics, offering high sensitivity and robust performance against environmental temperature variations.

## 4 Conclusion

This study developed a stretchable strain sensor that combined a high gauge factor and excellent linearity with temperature insensitivity under strain. This performance was achieved by employing a microcrack structure formed by stacking Ag NPs with DMSO‐doped PU‐PEDOT:PSS with near‐zero TCR. The significant resistance changes observed were attributed to the change in the current path from Ag to PU‐PEDOT:PSS caused by microcrack formation under applied strain. During elongation, the resistance of the sensor was insensitive to temperature, as the electrical properties of the PU‐PEDOT:PSS layer became dominant. This strategy effectively balances strain sensitivity with temperature insensitivity, enabling precise strain detection in wearable devices and robotics without interference from temperature fluctuations.

## 5 Experimental Section

### 5.1 Materials

PEDOT:PSS aqueous dispersion (CLEVIOS PH1000, Heraeus Deutschland GmbH & Co. KG) was filtered by a 0.45‐µm‐pore‐size filter. DMSO was added to PEDOT:PSS and stirred at room temperature for 30 min. Then, waterborne PU (SUPERFLEX E‐2000, DKS Co. Ltd.) filtered by a 5‐µm‐pore‐size filter was added to the ink and stirred at room temperature for 1.5 h. The mixing ratio of PU and PEDOT:PSS was 1:1.35 by weight of each dispersion; that is, the weight ratio of the solid component of PEDOT:PSS in PU‐PEDOT:PSS was approximately 3 wt%. DMSO concentration was defined as the weight concentration relative to the mixed ink of PU and PEDOT:PSS. For example, in the case of DMSO concentration of 10 wt%, PU, PEDOT:PSS, and DMSO were mixed in a weight ratio of 1:1.35:0.26, respectively. In this study, the DMSO concentration was 10 wt% unless otherwise specified. We used Ag NP inks (OAG‐AP028, Nagase ChemteX Corporation) that become conductive upon low‐temperature annealing at 80°C. To adjust the thickness of the Ag NP film, the Ag NP inks were mixed with chloroform in a 1:1 weight ratio and stirred at room temperature for 30 min.

### 5.2 Device Fabrication

Schematic and photographs of device fabrication are shown in Figure S1. A bank made of resin with a size of 4.5 cm × 6.0 cm was formed by 3D printing on a glass substrate. A release agent (Ease Release 205, Smooth‐On, Inc.) was drop‐casted onto the glass and annealed at 100°C for 10 min. 2 g of silicone elastomer (SILPOT 184, Dow Toray Co., Ltd.), which was a mixture of base and curing agent in a weight ratio of 20:1, was drop‐casted into the bank and annealed at 100°C for 1 h. The thickness of the silicone elastomer was approximately 550 µm. The cured silicone elastomer was then peeled off from the glass substrate and attached to another glass substrate. The surface was treated with oxygen plasma at 150 W for 10 min using a plasma cleaner (PC‐300, Samco Inc.), followed by the spin coating of PU‐PEDOT:PSS ink, Ag NP ink, and PU‐PEDOT:PSS ink at 1000 rpm in a dry chamber with humidity less than 25% to form a multilayer structure. After each spin‐coating process, the sample was annealed in air at 120°C for 10 min. Finally, the sample was peeled off from the glass substrate and cut into an ISO 37‐4 dumbbell shape using a sample cutter (SDMP‐1000, DUMBBELL CO., LTD.). For comparison with the multilayer structure, we also fabricated single‐layer structures in which only PU‐PEDOT:PSS or Ag NPs were coated on a silicone elastomer (Figure S5).

### 5.3 Electrical Characterization

A precision universal testing machine (AG‐X, Shimadzu Corporation) was used for the dynamic strain evaluation, in which the strain was changed continuously. Films with Cu electrodes were attached to the device, and the device with the electrodes was clamped in grips. Resistance was measured using multimeters (34465A and 34470A, Keysight Technologies Inc.) by the four‐terminal method. The initial distance between the grips in the unstrained state was 18 mm. The strain during extension was calculated from the grip distance. The speed of extension and contraction was 0.9 mm s^−1^ in the durability measurement and 0.3 mm s^−1^ in the other measurements. A thermostatic chamber (TCE‐N300A, Shimadzu Corporation) attached to the precision universal testing machine was used for temperature control. A thermocouple was placed near the device to monitor the temperature. The temperature was controlled for the evaluation of the temperature dependence of devices, and measurements were performed at room temperature (22°C–25°C) for other evaluations.

A linear stage was used for TCR measurement under static strain. Wiring was attached to the device with Ag paste, followed by annealing at 120°C for 5 min. Both ends of the device were fixed to the linear stage using acrylic tape. The initial distance between the stages in the unstrained state was 15 mm. Strain was controlled by adjusting the distance between the stages. The linear stage was set in an oven (DKN302, Yamato Scientific Co., Ltd.), and the temperature was controlled. A thermocouple was placed near the device to monitor the temperature. Resistance was measured using multimeters (34465A and 34470A, Keysight Technologies Inc.) by the four‐terminal method. Samples were heated to 70°C with the strain fixed before the measurement.

### 5.4 Analysis of Structures

An optical microscope (VHX‐7100, KEYENCE CORPORATION) was used to observe microcracks in devices. The device was mounted on a linear stage, and strain was controlled by adjusting the distance between the stages. Optical microscopic images were captured from the top surface.

For cross‐sectional analysis, the surface of the device was coated with a protective film, and a focused ion beam was used to vertically etch the device and expose its cross‐section. Low‐magnification images, covering the entire multilayer structure, were obtained using a SEM (Helios G4, Thermo Fisher Scientific Inc.) with a tilt angle of 52° and an acceleration voltage of 1 kV. To analyze the details of the PU‐PEDOT:PSS layer, high‐magnification images were obtained using a SEM (Crossbeam 550, Carl Zeiss Co., Ltd.) at a tilt angle of 54° and an acceleration voltage of 5 kV. Elemental mapping of sulfur was obtained using EDX (Ultim Max 170, Oxford Instruments plc) at the same field of view and acceleration voltage as the high‐magnification SEM image.

## Conflicts of Interest

The authors declare no conflicts of interest.

## Supporting information




**Supporting File**: advs73912‐sup‐0001‐SuppMat.docx.

## Data Availability

The data that support the findings of this study are available from the corresponding author upon reasonable request.
