# Supplementary material for: Highly Sensitive and Temperature‐Insensitive Stretchable Strain Sensor with Microcrack‐Engineered Architecture
Source: Adv Sci (Weinh). 2026 Jan 18;13(19):e21634. doi: 10.1002/advs.202521634 (PMC13045208; doi:10.1002/advs.202521634)
Supplement: Supplementary file 1 — Supporting File: advs73912‐sup‐0001‐SuppMat.docx. [file ADVS-13-e21634-s001.docx]

**Supporting Information**

**Highly Sensitive and Temperature-Insensitive Stretchable Strain Sensor with Microcrack-Engineered Architecture**

Yu Kato,^1^ Kento Yamagishi,^1^ Kenjiro Fukuda,^2,3,+^ Takao Someya^1,2,3^* and Tomoyuki Yokota^1,4^*

*^1^Department of Electrical Engineering and Information Systems, The University of Tokyo, 7-3-1 Hongo, Bunkyo-ku, Tokyo 113-8656, Japan*

*^2^ RIKEN Center for Emergent Matter Science, 2-1 Hirosawa, Wako, Saitama 351-0198, Japan*

*^3^Thin-Film Device Laboratory, RIKEN, 2-1 Hirosawa, Wako, Saitama 351-0198, Japan*

*^4^Institute of Engineering Innovation, Graduate School of Engineering, The University of Tokyo, 7-3-1 Bunkyo-ku, Tokyo, 113-8656, Japan*

*+current address: Graduate School of Engineering, The University of Osaka, 2-1 Yamadaoka, Suita, Osaka, 565-0871, Japan*

**Corresponding author. E-mail: someya@ee.t.u-tokyo.ac.jp (T.S.);* *yokota@ntech.t.u-tokyo.ac.jp (T.Y.)*

**Table S1.** Summary of the performance of temperature-insensitive stretchable strain sensors with stretchability of over 10%.

| Ref. | Sensor material  (Structure) | Max.  strain | Max.  GF | Linearity and GF in the range from 0% to a certain strain | Absolute value of TCR (% K^−1^) | | Effect of temperature on resistance under cyclic strain* |
| --- | --- | --- | --- | --- | --- | --- | --- |
|  |  |  |  |  | in the initial state | under static strain |  |
| [51] | WCNTs, FSE | 30% | ~2 | *R*^2^: unclear, GF: ~2  (*ε*: 30%) | 0.0026 | No data | Small (*ε*: 30%, −20–25 °C)  Medium (*ε*: 30%, 25–100 °C) |
| [41] | Graphene,  Ag NWs, PDMS | 47% | 9156  (−40 °C) | *R*^2^: unclear, GF: 80  (*ε*: 24%) | 7.04×10^−3^ | No data | Large (*ε*: 40%, −40–20 °C)  Small (*ε*: 20%, −40–45 °C) |
| [52] | CNTs, GNPs  (Sponge) | 60% | 43000 | *R*^2^: 0.98, GF: 2393  (*ε*: 25%) | Unclear | No data | No data |
| [40] | CNTs, GNPs,  PDMS | 80% | 51.4  (−20 °C) | *R*^2^: 0.974, GF: 10.6  (*ε*: 40%, −20 °C) | ≤0.243 | No data | Medium (*ε*: 40%, −20–40 °C) |
| [53] | GrF, Ag NTs,  Silicone (Foam) | 90% | 227 | *R*^2^: 0.97, GF: 2.9  (*ε*: 40%) | 0.29 | No data | Small (*ε*: 50%, 15–45 °C) |
| [39] | CNTs, GNPs,  Silicone | 100% | 14550.2 | *R*^2^: unclear, GF: 10.7  (*ε*: 40%) | 1.14×10^−2^ | No data | Medium (*ε*: 20%, 25–60 °C) |
| [38] | Ag NPs, CNTs  (Mesh shape) | 100% | 326.5  (−40 °C) | *R*^2^: 0.960, GF: 38.2  (*ε*: 40%, −40 °C) | 1.16×10^−3^ | No data | Large (*ε*: 50%, −40–20 °C)  Small (*ε*: 50%, 20–60 °C) |
| [29] | EGaInSn | 140% | 3.4 | *R*^2^: unclear, GF: 2.2–3.4  (*ε*: 140%) | Unclear | No data | No data |
| [10] | Ag NPs, ACNTs, FCNTs-SiO_2_ (Fabric) | 155% | 6.6×10^4^ | *R*^2^: 0.95, GF: 10.83  (*ε*: 5%) | Unclear | No data | Small (*ε*: small, −40–45 °C) |
| [54] | Graphene  (Kirigami structure) | 240% | <1 | *R*^2^: unclear, GF: <1  (*ε*: 130%) | Unclear | No data | No data |
| [16] | EGaIn | 320% | 4.91 | *R*^2^: unclear, GF: 1.5–4.91  (*ε*: 320%) | Unclear | No data | No data |
| [50] | SiO_2_ NPs-PAAm-PPy hydrogel | 500% | 2.90 | *R*^2^: unclear, GF: 1.70  (*ε*: 250%) | Unclear | No data | Small (*ε*: 100%, −20–25 °C) |
| [2] | PAM/Bet-Gly eutectogel | 500% | 14.32 | *R*^2^: unclear, GF: 1.66  (*ε*: 150%) | Unclear | No data | Small (*ε*: 100%, −80–80 °C) |
| **This study** | **PU-PEDOT:PSS,**  **Ag NPs**  **(Multilayer)** | **200%** | **2.3×10^4^** | ***R*^2^: 0.98, GF: 2.3×10^4^**  **(*ε*: 50%)** | **2.3** | **<0.1**  **(*ε*: 5–50%)** | **Small: 7%**  **(*ε*: 50%, 25–65 °C)** |

*Difference in peak resistance under cyclic strain between different temperatures was estimated from the figures in the references. Less than 20% for “small”, 20% to 100% for “medium”, and more than 100% for “large”.

(Abbreviations)

GF: gauge factor, TCR: temperature coefficient of resistance, WCNT: whisker CNTs, CNTs: carbon nanotubes, FSE: fluorosilicone elastomer, NWs: nanowires, PDMS: polydimethyl siloxane, GNPs: graphene nanoplatelet, GrF: graphene foam, NTs: nanostructures, NPs, nanoparticles, EGaInSn: eutectic gallium indium tin, ACNTs: acid-modified CNTs, FCNTs: perfluorodecyltriethoxysilane modified CNTs, EGaIn: eutectic gallium indium, PAAm: polyacrylamide, PPy: polypyrrole, PAM: polyacrylamide, Bet: betaine, Gly: glycerol, PU: polyurethane, PEDOT: poly(3,4-ethylenedioxythiophene), PSS: poly(4-styrenesulfonate)

**Figure S1.** Schematic cross-section and photographs of device fabrication.

**Figure S2.** Resistance and conductivity of PU-PEDOT:PSS single-layer structures in the unstrained state as a function of DMSO concentrations. The error bars in the plots indicate standard deviations based on seven devices for 10 wt% DMSO and three devices for the other conditions.

**Figure S3.** Analysis of microcracks in a multilayer strain sensor. a) Optical microscopic images at different strains. Strain was applied horizontally in the images. 50% strain was applied to the device prior to the analysis to form microcracks. b) Length of microcracks as a function of strain. The measured microcracks correspond to the microcracks identified by letters in the image on the right.

**Figure S4.** Cross-sectional analysis of a multilayer strain sensor. a) SEM image of the sensor. b) Magnified SEM image of PU-PEDOT:PSS layer. c) EDX elemental mapping of sulfur observed in the same area as (b).

**Figure S5.** Comparison of fabricated device structures.

**Figure S6.** Resistance of devices with different structures in the unstrained state. The error bars in the plots indicate standard deviations based on seven devices.

**Figure S7.** Characteristics of seven multilayer strain sensors.

**Figure S8.** Response and recovery times of multilayer strain sensors. a) Response time. b) Recovery time. The inset shows a magnified view of the vertical axis.

**Figure S9.** Durability of the multilayer strain sensor under cyclic strains ranging from 0% to 50% for approximately 1000 cycles at room temperature. Relative resistance change in the initial cycles (a), after 100 cycles (b), after 1000 cycles (c) and after 1000 cycles and stabilization (d). d) 50% strain was applied at 70 °C as the stabilization process.

**Figure S10.** Characteristics of the multilayer strain sensor without DMSO at different temperatures under cyclic strain. a) Relative resistance change for 10 cycles of 50% cyclic strain at each temperature. b,c) Relative resistance change as a function of strain. Figures with linear scale (b) and logarithmic scale (c) on the vertical axis. a-c) *R*_0_ denotes the resistance of the device without strain at 25 °C. 50% strain was applied at 70 °C before the evaluation.
